# The Inhibition of Adipose-Derived Stem Cells on the Invasion of Keloid Fibroblasts

**DOI:** 10.7150/ijms.68646

**Published:** 2022-10-03

**Authors:** Jiong Zhou, Ji-Yang Shen, Li-En Tao, Huan Chen

**Affiliations:** 1 Department of Dermatology, 2nd Affiliated Hospital, School of Medicine, Zhejiang University, Hangzhou, Zhejiang, China.; 2 Department of Dermatology, Ningbo Medical Center Lihuili Hospital, Ningbo, Zhejiang, China.; 3 Department of Dermatology, The Central Hospital of Lishui City, Lishui, Zhejiang, China.

**Keywords:** keloids, adipose-derived stem cells, fibroblasts, collagen, wound healing

## Abstract

**Background:** Keloids represent the dysregulation of cutaneous wound healing caused by aberrant fibroblast activities. Adipose-derived stem cells have been recognized as a promising treatment for keloids. However, the molecular mechanisms have not been fully elucidated.

**Objectives:** to explicitly demonstrate the relationship between adipose-derived stem cells alleviating keloids and alterations of Col-1, Col-3, CTGF, and P-4-HB.

**Methods:** Skin biopsies were obtained from 10 keloid patients and 9 healthy volunteers. Fibroblasts isolated from all samples were divided into two groups, one co-cultured with adipose-derived stem cells and the other grown independently. We compared the wound-healing rates, fibroblast survival rates, apoptosis rates, mRNA expressions, and protein levels of Col-1, Col-3, CTGF, and P-4-HB between separated groups.

**Results:** We found no significant differences between normal fibroblasts and keloid fibroblasts in terms of wound-healing rate, survival rate, or apoptosis rate at the baseline. With adipose-derived stem cells, wound-healing rate and survival rate of normal fibroblasts were promoted, whereas in keloid fibroblasts, they were reduced. The apoptosis rate of normal fibroblasts and keloid fibroblasts were restrained, with the restraint in keloid fibroblasts being more evident. The protein levels of Col-3, CTGF, and P-4-HB were lower in keloid fibroblasts co-cultured with adipose-derived stem cells than in normal fibroblasts under similar conditions.

**Conclusions:** Adipose-derived stem cells strongly suppressed keloid fibroblasts' proliferative and invasive behavior. However, adipose-derived stem cells negatively regulated keloid fibroblast apoptosis. Adipose-derived stem cells can be a potential keloid therapy worth further investigation.

## Introduction

Keloids refers to dysregulation of cutaneous wound healing, which may develop up to several years after minor injuries, such as trauma, burns, and surgery, even without the patient's awareness. Keloids exceed the wound border, lack natural regression, and are accompanied by itch and pain. Therefore, patients suffer from substantial physical and psychological distress.

Management for keloids include silicone, corticosteroids, botulinum toxin A, pulsed-dye laser, CO_2_ laser, 5-fluorouracil, bleaomycin, surgery, radiation therapy, interferon, interleukin-10, and adipose-derived stem cells (ASCs) 1. ADSCs have been recognized to impact wound healing, soft-tissue restoration, and scar remodeling, due to their angiogenic and anti-apoptotic properties 2. However, the molecular mechanism of ASCs in treating keloid remains unclear.

In the dermis, fibroblasts (FBs) are the key cells of skin collagen synthesis. Collagen type I (Col-1, 80%, encoded by gene Col1A1) and type III (Col-3, 20%, encoded by gene Col3A1) are the two leading collagen compositions 3.

Histologically, multiform, thicker collagen fibers are stacked tightly in keloids. Compared to normal skin, Col-3 is over-synthesized, whereas Col-1 levels are reduced 4. Unshaped extracellular matrix (ECM) surround FBs in the absence of appendages.

Several signal pathways have been found to be involved in keloid formation. To date, studies on how transforming growth factor (TGF)-β1/Smad3 signaling pathways interact with keloid are the most detailed, in which TGF-β1 plays an important role in keloid formation. Its effects include chemokine secretion to recruit inflammatory cells and FBs to the wound site 5, stimulation of collagen synthesis, and suppression of collagen degradation 6. TGF-β1 also induces autocrine of connective tissue growth factor (CTGF) in keloid FBs 7.

Prolyl 4-hydroxylase B (P-4-HB) is necessary for the stabilization of the collagen triple helix structure, enhancing the effect of collagen crossing and scarring 8.

In recent years, it has been discovered that ASCs aid in reversing keloid formation 9-11. However, the mechanisms have not been fully elucidated. Therefore, our research was conducted to explicitly describe the pathways associated with ASCs' alleviation of keloids.

## Materials and Methods

### Sample collection, primary FBs culture, and reagents

The study was approved by the ethics committee of the 2nd Affiliated Hospital, School of Medicine, Zhejiang University. Skin samples were obtained from 10 keloid patients and 9 healthy volunteers (9 males and 10 females, 18-55 years old, samples of 3 keloid patients and 3healthy volunteers are used in cell experiments) at cosmetic surgical operations (face or chest) after signing informed consents.

Samples were placed in 0.5% dispase (Invitrogen, Carlsbad, CA) at 4 °C overnight, and the dermis was separated from the epidermis after overnight incubation with 0.1% collagenase (Invitrogen). The released FBs were cultured in DMEM containing 10% fetal bovine serum. FBs between the second and the sixth passages were used in subsequent experiments. FBs from healthy volunteers were sorted into Groups F1, F2, and F3. FBs from keloid tissues were grouped into F4, F5, and F6 12.

### Adipose-derived stem cells isolation and identification by flow cytometry

All human adipose tissues were from three volunteers (1 male and 2 females) without systemic diseases, aged 20 to 25 years old. They underwent liposuction surgery after providing their informed consent and with the due approval of the Research Ethical Committee of the 2nd Affiliated Hospital, School of Medicine, Zhejiang University.

Fifty ml human lipoaspirate was washed three times in phosphate-buffered saline (PBS; Hyclone, USA) and finely minced, before being digested in 0.1% collagenase I (Sigma, USA) at 37.0 °C with continuous vibration for approximately 1 hour. Digestion was terminated by adding Dulbecco's Modified Eagle Medium/F12 (DMEM; Hyclone, USA) containing 10% Mesenclaymal stem cell Bovine Serum (MFBS; BI, USA). The tissue was centrifuged at 1,200 revolutions per minute for 5 min, at the temperature of 25°C. Then, the supernatant was discarded, and the cell pellet was washed in PBS before being centrifuged at 1,000 revolutions per minute for 5 min, at the temperature of 25°C. Cells were resuspended in common culture media (DMEM containing 10% MFBS) and counted; then, they were inoculated at a density of 1×10^9^ cells per liter. The first culture medium change was conducted 24 h after inoculating and was routinely repeated every 72 h. When cells reached 80% to 90% confluence, the subculture was operated using 0.25% trypsin with 0.03% EDTA (Gibco, USA). Human ASCs (hASCs) between the third and the fourth passages were used in the subsequent experiments.

hASCs (passage 2) were harvested and washed in PBS three times. Cell suspension was incubated with FITC-conjugated antibodies against CD45 (11-0459-42, eBioscience), CD34 (22-0349-42, eBioscience), PE-conjugated antibodies against CD105 (12-1057-42, eBioscience), PE Cyanine5-conjugated antibodies against HLA-DR (11-0459-42, eBioscience) at 37.0 °C for 15 min in the dark, followed by washing and resuspension in PBS. Then, it was finally detected by flow cytometer Model Number ACCURI C6 (BD Biosciences, USA) and supporting software BD Accuri C6 Software (BD Biosciences, USA).

### Immunohistochemistry analysis

The paraffin-embedded samples were sliced into 4-μm sections and incubated with primary antibodies: Anti-Col-1 (ab34710, Abcam. Dilution 1:100), Anti-Col-3 (ab7778, Abcam. Dilution 1:100), Anti-CTGF (ab6992, Abcam. Dilution 1:100), and Anti-P4HA2 (ab211527, Abcam. Dilution 1:100) after dewaxing, hydration, antigen retrieval, and blocking. The samples were observed under microscope the Olympus BX43. The expression was detected using the immunoperoxidase technique (DAB kit, China).

Every slide was observed 5 visions randomly under microscope, at magnification of 20×.

The expression of Col-3, P-4-HB, Col-1, CTGF was measured by semi-quantitative analysis. The stain intensity equaled to cell stain intensity minus background stain intensity. No staining was graded 0. Slight staining was graded 1. Medium staining was graded 2. Heavy staining was graded 3. Percentage of positive cells was calculated on every 5 visions (20×).

All the IHC slides were graded from 0-4. Grading was based on the percentage of positive cells with IHC nuclear labeling: 0-5% labeling was graded 0, 6-25% labeling was graded 1, 26-50% was graded 2, 51-75% was graded 3, and 76-100% labeling was graded 4.

Finally, we summed up stain intensity grading and positive cell percentage grading of every vision. We assigned criteria of negative, intermediate and positive as follows: a grade of 0 was considered negative, 1-3+ was considered slight positive, 4-5+was considered moderate positive, 6-7 was considered high positive.

### Quantitative real-time PCR

Total RNA was extracted from nFBs and kFBs using TRIzol reagent (Generay Biotech, Shanghai, Cbina), according to the manufacturer's protocol. Col1A1, Col3A1, CTGF, P-4-HB, and β-actin mRNA were analyzed by quantitative real-time PCR. The primers for Col1A1 were AAGGTGTTGTGCGATGACG and TGGTCGGTGGGTGACTCTG. The primers for Col3A1 were CCCGTATTATGGAGATGAAC and ATCAGGACTAATGAGGCTTTC. The primers for CTGF were ACCCAACTATGATTAGAGCC and TTGCCCTTCTTAATGTTCTC. The primers for P-4-HB were AGGTTCCGAGATCAGGTTG and GGAAGCCGTGTCTCCATT. A total of 2 μg of Trizol-isolated total RNA was DNase treated, and cDNA was synthesized by using Superscript II Reverse Transcriptase (Invitrogen). Gene expression was determined by using SYBR Green PCR mix (TaKaRa, Dalian, China) and 10 ng of template. Real-time PCR was performed on ABI StepOne Plus instrument, using the following amplification conditions: 10 s at 95 °C; followed by 40 cycles of 10 s at 95 °C; 20 s at 60 °C, and 20 s at 72 °C. CT values were analyzed with qBase Plus 2 software (Biogazelle, Zwijnaarde, Belgium) by 2^-△△CT^ calculation.

### Co-culture of FBs and ASC, CCK8 assay for cell growth curve

Normal FBs (F1, F2, and F3) and keloid FBs (F4, F5, and F6) were co-cultured with ASCs (grouped into A1, A2, and A3) in a 24-well plate (Table 1). The FB suspension was seeded in a 24-well plate (5×10^4^/well). Then, ASCs (5×10^4^/well) were plated onto the chamber (0.4 μM). The co-culture lasted for 48 h when 10% CCK8 solution was added to each well, and the plates were incubated for 1h. Finally, the absorbance was measured at 450 nm using a microplate reader.

### Cell apoptosis detection

After 48 h of co-culture with ASCs, FBs were collected and resuspended in 1× binding buffer at a concentration of 1.0 × 10^6^ cells/mL, and 100 µL of sample was mixed with 5 µL of FITC Annexin V (AP105-60-kit provided by MULTISCIENCES BIOTECH, CO., LTD, Hangzhou, China) and 5 µL of propidium iodide (PI). The sample was mixed gently and shielded from light at room temperature for 5 min. Then, 400 µL of 1× binding buffer was added. The sample was detected and analyzed within 1 h by flow cytometry.

### *In vitro* wound healing assay

nFBs (F1, F2, F3) and kFBs (F4, F5, F6) were plated to create a confluent monolayer onto a six-well chamber with transwell. The ADSCs were seeded on insert. The number of FBs in each well is 2×10^5^. The number of ASCs in each well is 1×10^5^. After being grouped into normal control and co-cultured with A1, A2 or A3, scratched wounds were created by using a sterile p200 pipet tip. Cells migrating into the wound space were estimated at 0 h and 24 h after wound with image analysis. Wound closure was determined as the difference between wound areas at different time point. At least 3 independent experiments were carried out in triplicate.

### Western blot analysis

Equal amount of cell lysates from each sample was first separated using 7.5% SDS-PAGE, and then transferred onto nitrocellulose membranes (Millipore, Bedford, MA). After being blocked with 7% nonfat dry milk in Tris-buffered saline-Tween at room temperature for 1 hour, the membranes were probed with specific primary antibodies, followed by incubation with horseradish-peroxidase-conjugated anti-rabbit, or anti-mouse secondary antibodies. Chemiluminescence Reagent Plus (Millipore) was used to develop images. β-actin was measured as a loading control.

Antibodies against Col-1, Col-3, CTGF, and P-4-HB were purchased from Abcam, and antibodies against β-actin were purchased from Multi Sciences.

### Statistical analysis

All of the experiments were performed in triplicate at minimum. Data were presented as the mean ± standard deviations from more than two independent experiments.

The Kolmogorov-Smirnov test using SPSS 16.0 (SPSS, Inc) software was used to determine whether or not data fitted normal distribution. GraphPad Prism 7 (GraphPad Software, USA) software was used to evaluate statistically significant differences by *t*-test and to generate graphs. Results were considered statistically significant when the probability was less than 5% (p < 0.05).

## Results

### Characterization and identification of hASC

Human adipose-derived stem cells (hASCs) were adherent to plastic and demonstrated FB-like morphology after two passages (Supplementary Figure 1). Immunophenotype was assessed, and results revealed that the cells were positive for MSC marker CD105 and negative for HLA-DR, CD45, CD34, according to the international criteria for defining multipotent MSC 13 (Supplementary Figure 2).

### hASCs enhance the function of nFBs more than kFBs in wound scratch *in vitro*

**Figure 1:**
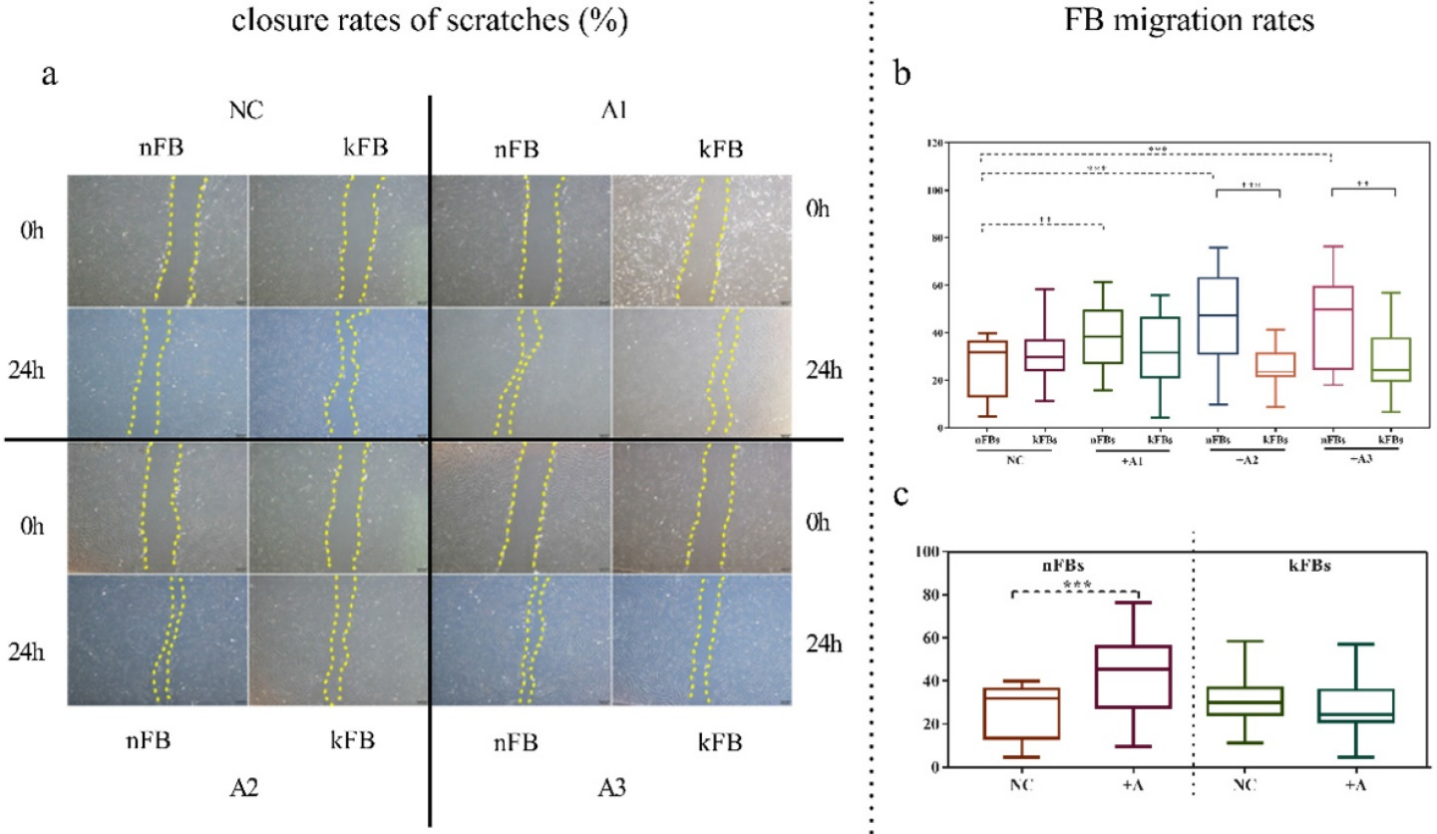
** Comparison of closure rates of scratches and FB migration rates. a.** In co-cultural groups with A2 and A3, the scratch closure rates of nFBs were significantly higher than that of kFBs after 24-h co-culture. **b and c.** The migration rates of three strains of nFBs in co-cultured groups were higher than those of nFBs in the NC group. *p < 0.05; **p < 0.005; ***p < 0.001. NC: normal control. nFBs: normal fibroblasts; kFBs: keloid fibroblasts. A: adipose-derived stem cells.

FB migration rate was compared using scratch tests (Figure 1). The artificial scratch was closed by proliferation and migration of the cultured cells. At the baseline, keloid FBs displayed better migration capability compared to normal FBs. ASC promoted closure of scratches in normal FBs by 43.3% (± 15.3); however, the cells restrained the migration of keloid FBs by 28.1% (± 9.5) as compared with controls after 24 h (Figure 1a). In co-cultural groups with A2 and A3, the FB migration rates of normal FBs were significantly higher than that of keloid FBs. Groups co-cultured with A1 demonstrated a slight trend toward significance. In the normal control (NC) group, we observed no significance (Figure 1b). FB migration rates of normal FBs co-cultured with A1, A2, and A3 apparently surpassed those in the NC group (Figure 1c). Results revealed conclusively that hASCs enhanced the function of normal FBs more impressively compared to keloid FBs in wound scratch *in vitro*.

### hASCs improve the survival rate of nFBs more effectively than kFBs

**Figure 2:**
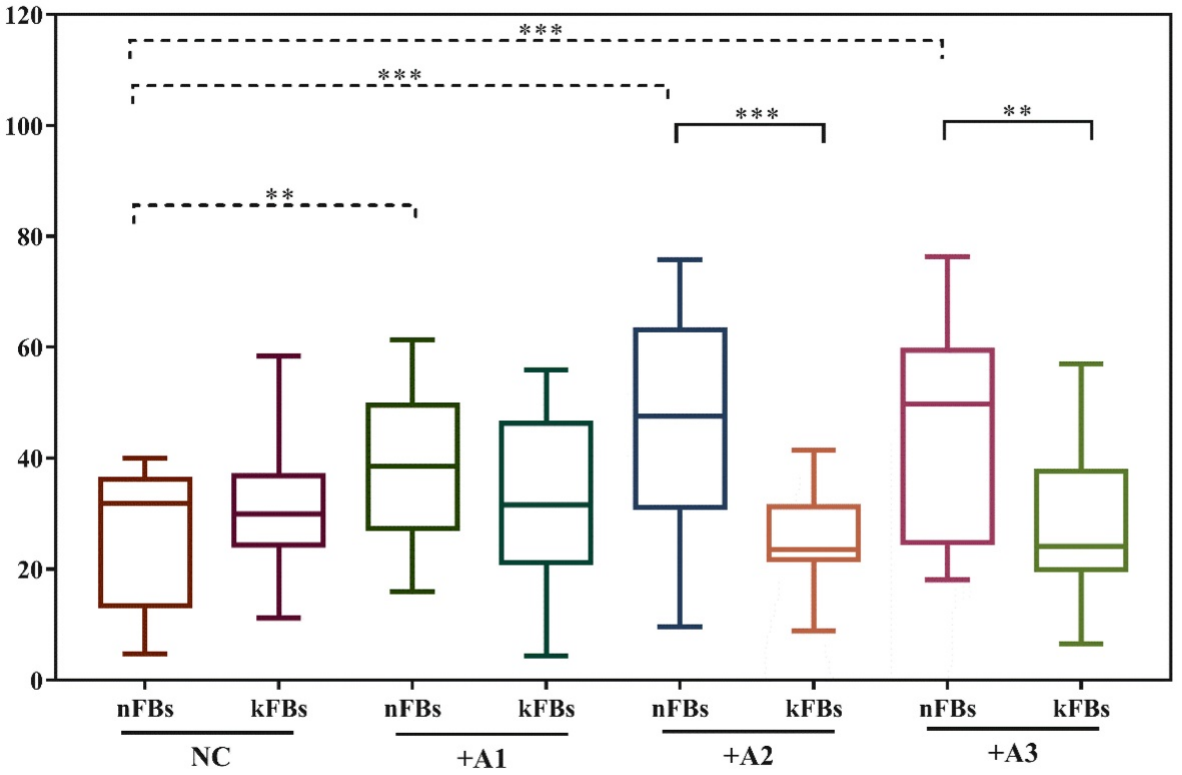
** Comparison of fibroblast survival rates.** There was no obvious difference between survival rates of normal and keloid FBs in the normal control group. However, the survival rate of nFBs was obviously higher than kFBs co-cultured with ADSC. The survival rates of three strains of kFBs were lower than that of kFBs in the normal control group. **p < 0.005. NC: normal control. A: adipose-derived stem cells. nFBs: normal fibroblasts; kFBs: keloid fibroblasts.

CCK8 tests were used to examine the cellular survival rate of normal and keloid FBs in the NC group or when co-cultured with ASCs (Figure 2). Comparison of cellular survival rates of normal keloid FBs in the NC group revealed no significant differences. However, in all three co-cultured groups, cellular survival rates were all obviously higher for normal FBs (p < 0.01). Compared to keloid FBs in the NC group, the survival rates of keloid FBs co-cultured with A1, A2, and A3 were all lower (p < 0.01). Such a result demonstrated that hASCs could effectively improve the survival rate of normal FBs and repress the survival of keloid FBs. Accordingly, it was necessary to investigate whether hASCs could induce apoptosis in keloid FBs rather than activating normal FBs.

### hASCs suppress keloid apoptosis

**Figure 3:**
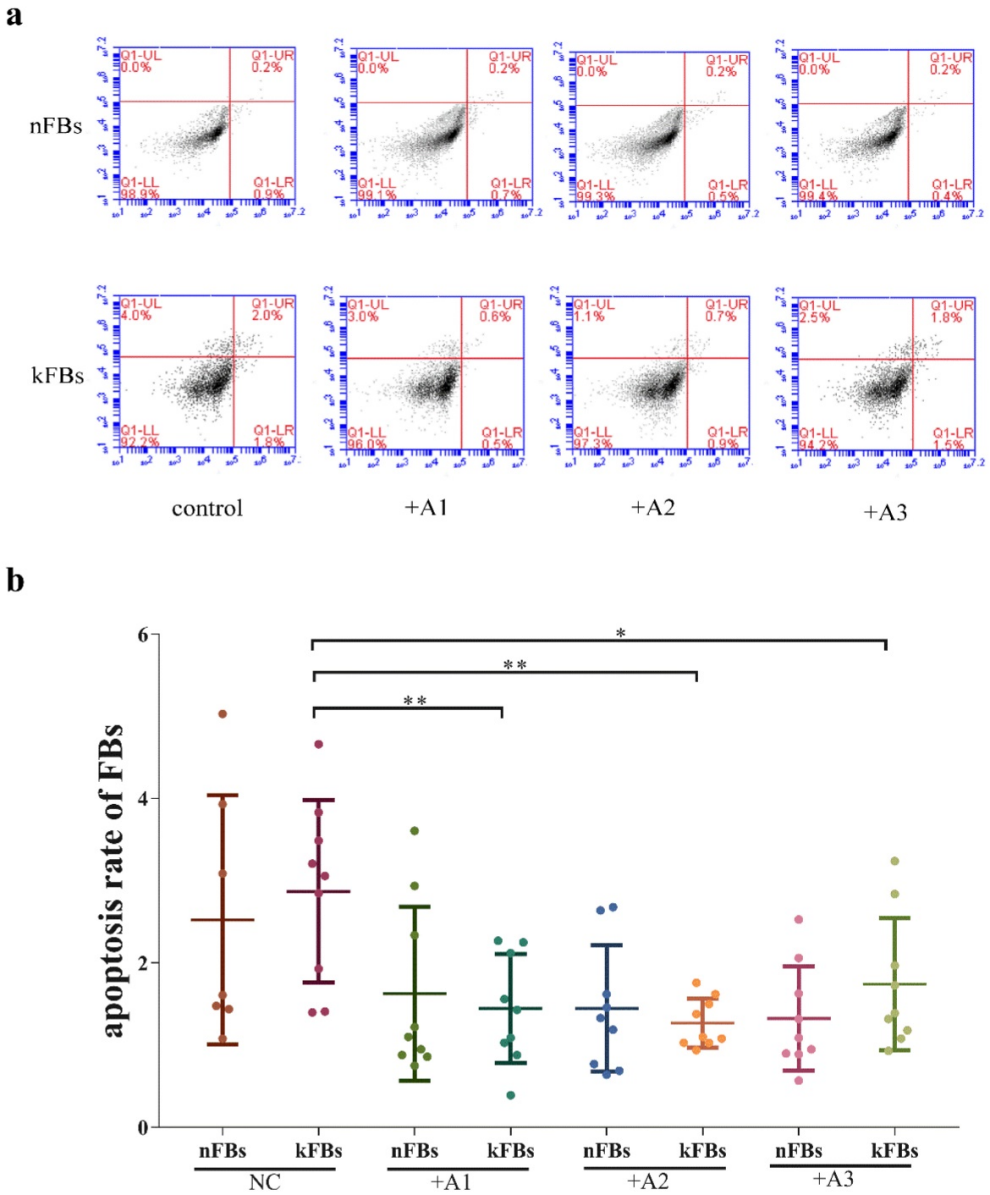
** Comparison of cellular apoptosis rates. a.** The flow cytometry graphs of co-cultural groups, corresponding to the columns in Graph b. **b.** The differences in cellular apoptosis rate between nFBs and kFBs were nonsignificant in all of the four groups, namely, normal control, A1, A2, and A3. However, apoptosis rates for kFBs in the three co-cultured groups were lower than that in the normal control group. *p < 0.05; **p < 0.005. NC: normal control. A: adipose-derived stem cells. nFBs: normal fibroblasts; kFBs: keloid fibroblasts.

In terms of apoptosis rate, the difference between normal FBs and keloid FBs was nonsignificant in all of the four groups (Figure 3). However, apoptosis rates of keloid FBs in three co-cultured groups were lower than those in the NC group. Based on the results of the CCK8 test, which suggested that hASCs could decrease keloid FB survival rates, there appeared to be a balance between hASCs' regulation of keloid FB survival and keloid FB apoptosis.

### mRNA expressions of Col-1, Col-3, CTGF, and P-4-HB in both nFBs and kFBs do not demonstrate much significance

**Figure 4:**
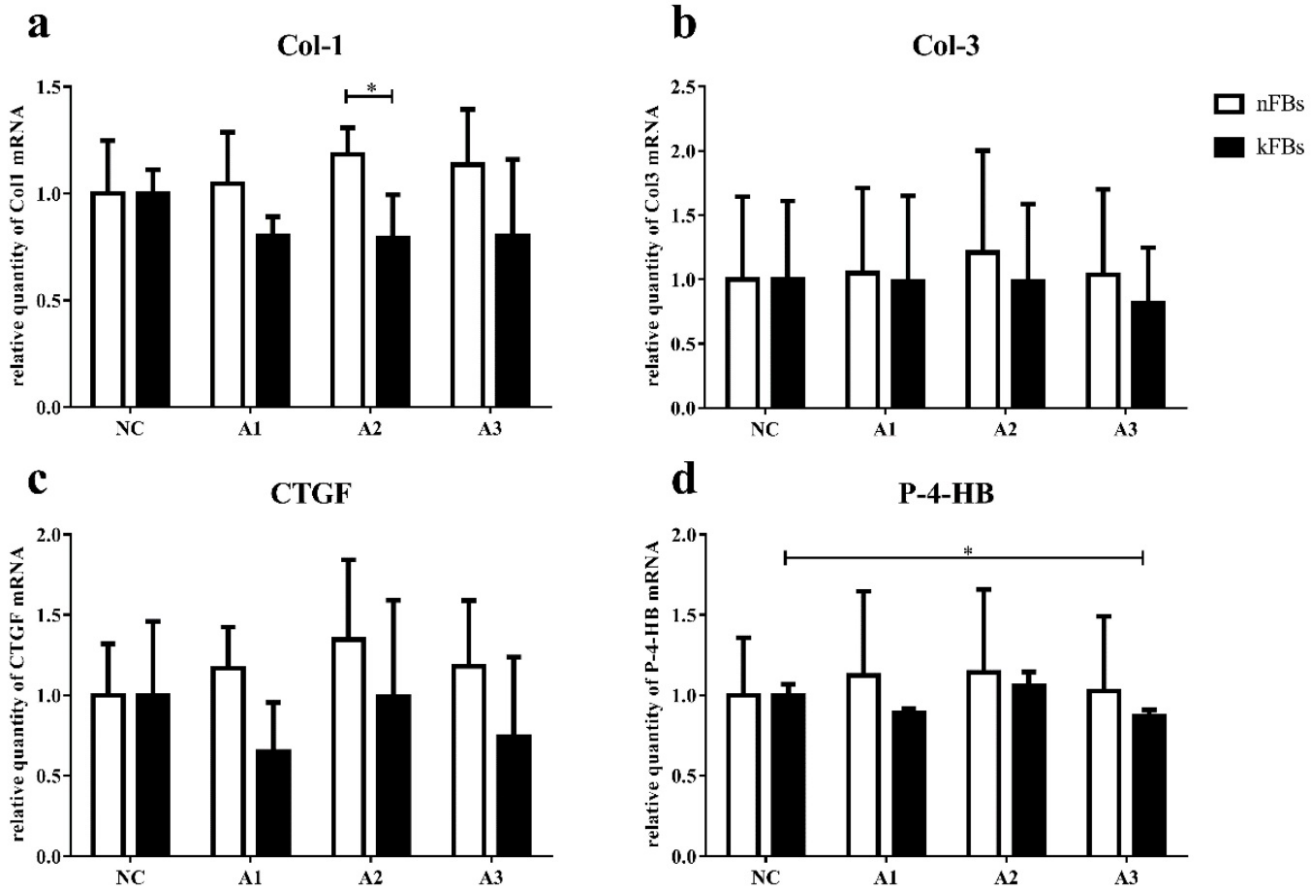
** mRNA expressions of Col-1, Col-3, CTGF, and P-4-HB. a.** The mRNA expression of Col-1 in kFBs was downregulated in co-cultural groups with A2 compared to nFBs. **d.** When co-cultured with A3, the P-4-HB expression was suppressed in kFBs, compared to kFBs in the NC group. *p < 0.05. A: adipose-derived stem cells. nFBs: normal fibroblasts; kFBs: keloid fibroblasts. NC: normal control. Col: collagen. CTGF: connective tissue growth factor. P-4-HB: Prolyl 4-hydroxylase B.

The mRNA level of Col-1, Col-3, CTGF, and P-4-HB in normal and keloid FBs did not display great significance in normal-control groups (Figure 4). In co-cultured groups, compared to normal FBs, keloid FBs appeared to be downregulated; however, only the A2 group demonstrated any significant difference.

When co-cultured with A3, P-4-HB expression was suppressed in keloid FBs, compared to keloid FBs in the NC group.

### Col-3 and P-4-HB were over-synthesized in keloid

**Figure 5:**
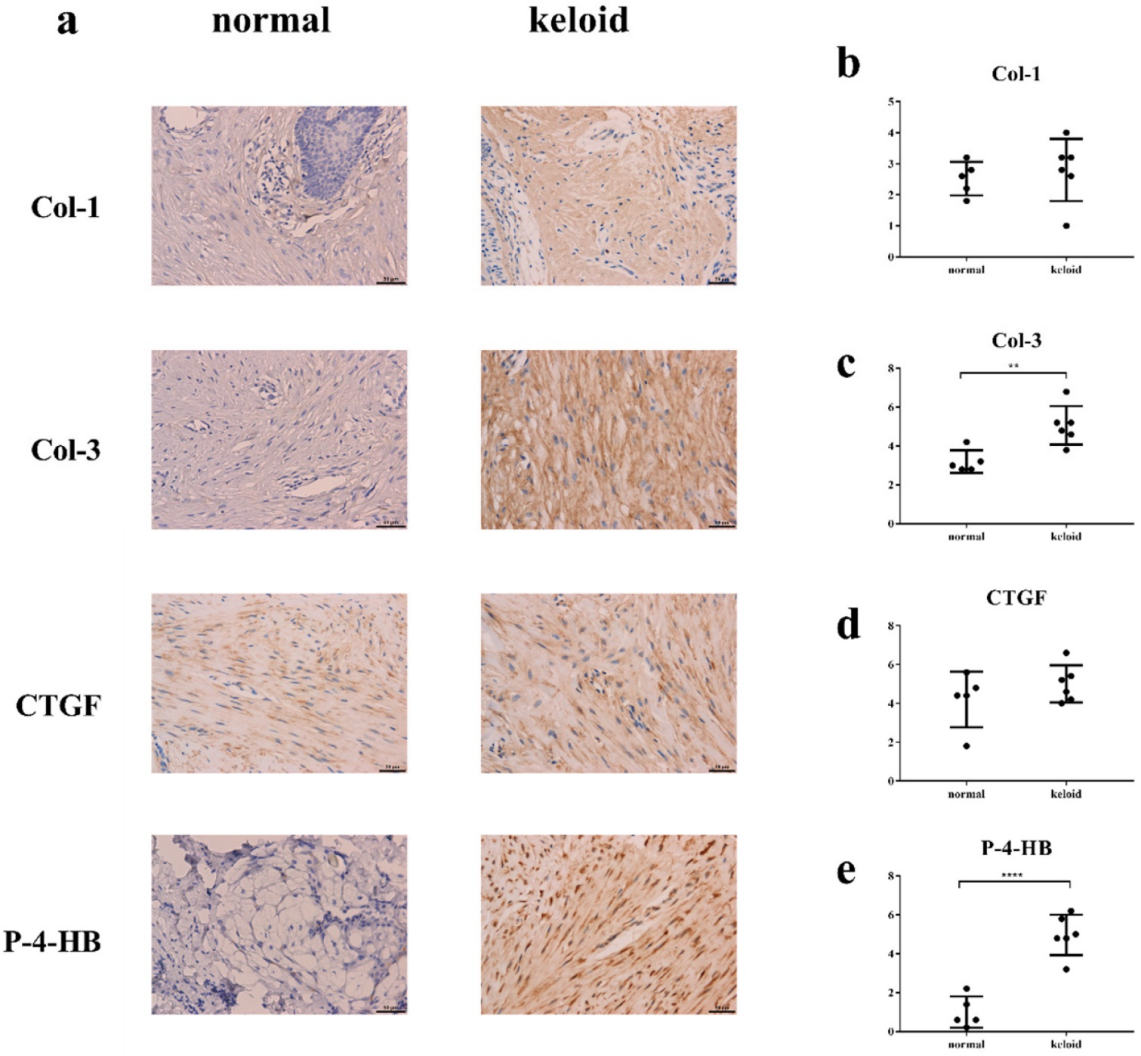
** The result of immunohistochemistry.** Y axis in Fig. 5b-e means sum of stain intensity grading and positive cell percentage grading of Col-1, Col-3, CTGF, P-4HB. **a, c, e.** The levels of Col-3 and P-4-HB were higher in keloid tissue than in normal tissue. **a, b, d.** The levels of Col-1 and CTGF were basically the same. **p < 0.005; ****p < 0.0001. Col: collagen. CTGF: connective tissue growth factor. P-4-HB: Prolyl 4-hydroxylase B.

Immunohistochemistry demonstrated that levels of Col-3 and P-4-HB were higher in keloid than in normal tissue (Figure 5). Levels of Col-1 and CTGF were basically the same.

### The protein levels of Col-3, CTGF, and P-4-HB are lower in kFBs co-cultured with hASCs than nFBs under the same condition

**Figure 6:**
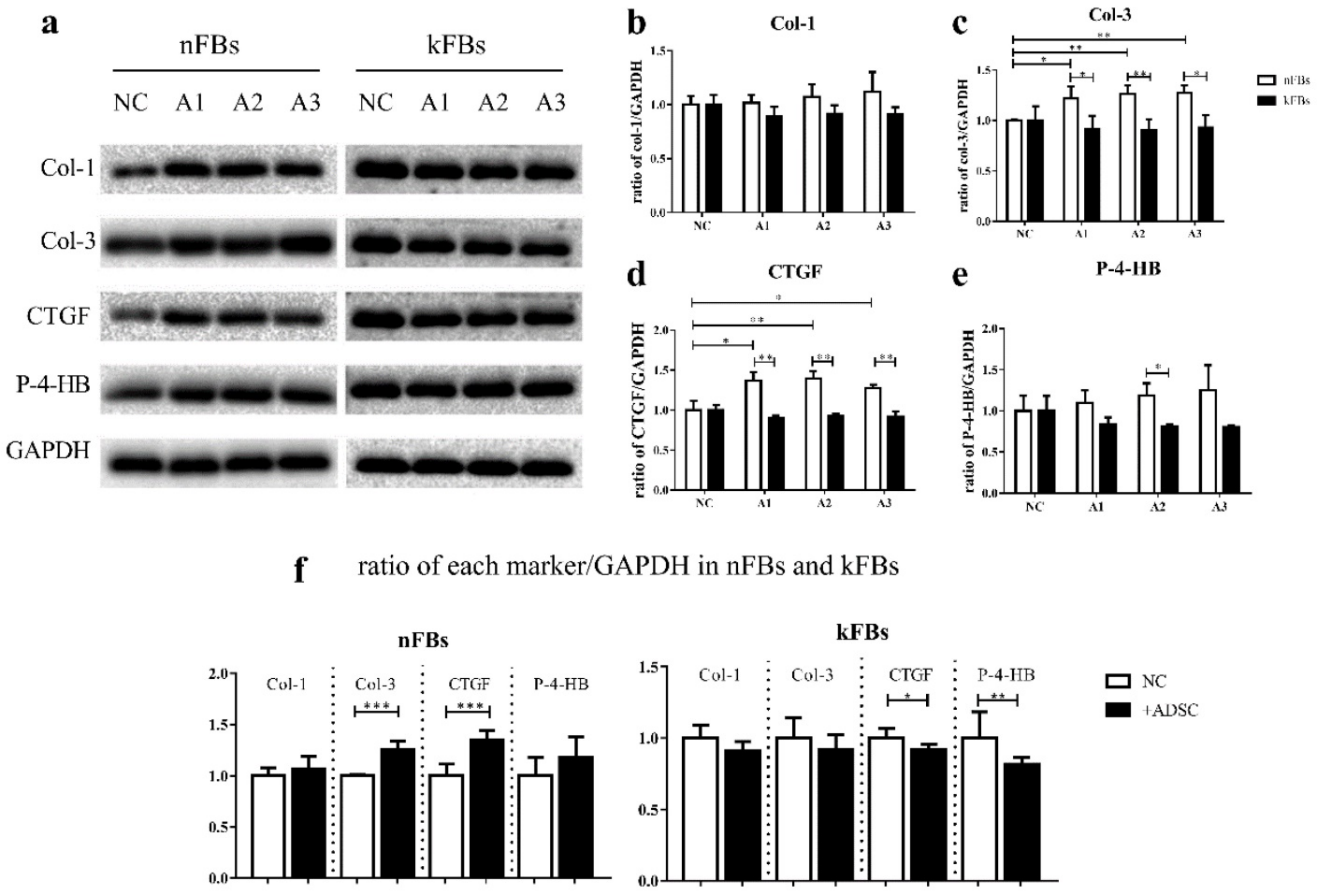
** Protein expressions of Col-1, Col-3, CTGF, and P-4-HB. a, c, d, e.** The protein levels of Col-3, CTGF, and P-4-HB were lower in kFBs co-cultured with ADSC than in nFBs under the same condition. a, b. The Col-1 protein level was not altered in co-cultured groups. f. The Col-3 and CTGF protein level of nFBs co-cultured with ADSC were higher than nFBs in NC groups. f. The CTGF and P-4-HB protein level of kFBs co-cultured with ADSC were lower than kFBs in NC groups.*p < 0.05; **p < 0.005; ***p < 0.001. A, ADSC: human adipose-derived stem cells. nFBs: normal fibroblasts; kFBs: keloid fibroblasts. NC: normal control. Col: collagen. CTGF: connective tissue growth factor. P-4-HB: Prolyl 4-hydroxylase B.

Western blot verified protein levels of Col-1, Col-3, CTGF, and P-4-HB in each group (Figure 6). The protein levels of Col-3 and P-4-HB were lower in keloid FBs co-cultured with hASCs than in normal FBs under the same condition. Furthermore, Col-1 protein levels were not altered. Results strongly demonstrated that hASCs can help suppress keloids by inhibiting Col-3 and P-4-HB production in keloid FBs. Intriguingly, in co-cultured groups, the CTGF protein level was also lower in keloid FBs than in normal FBs, which was not indicated by qRT-PCR.

With ASCs, the expression of Col-3, CTGF protein in normal FBs were upregulated. However, CTGF and P-4-HB displayed significant decrease in keloid FBs.

## Discussion

Keloids are human-specific pathological healing of skin lesions. The incidence of keloids in the Chinese population ranges between 4.6% and 16% 14. Keloid FBs are refractory to apoptosis. FBs located in the keloid margin are more highly proliferative than FBs in the center 4. Therefore, keloid borders develop beyond the original traumas.

Autologous fat transplantation has proven effective for pathologic scars both experimentally and clinically 15. However, the related molecular mechanisms are yet to be illustrated in detail. Further elucidation of the involved molecular mechanisms would confirm its effectiveness and even help develop potential therapies.

Wang's team 16 revealed that ASC-CM attenuated the bioactivities of keloid FBs by downregulating extracellular matrix-related gene expression, including plasminogen activator inhibitor-1, tissue inhibitor of metalloproteinase-1, and Col-1, and by inhibiting the expression of cell proliferation proteins. However, their research did not include comparisons with normal FBs.

In our study, we studied both normal and keloid FBs with hASCs, and duplicated these experiments with three different strains of hASCs, normal FBs, and keloid FBs. Analyses were conducted independently for each co-cultured group and for the NC group. Our results uncovered no significant differences between normal FBs and keloid FBs in terms of wound-healing rate, survival rate, or apoptosis rate at the baseline. Interestingly, with ASC, wound-healing rate and survival rate of normal FBs were promoted, while keloid FBs reduced evidently although the characterization of keloid FBs is high capacity of proliferation and aggressive properties 17. Both normal FBs and keloid FBs exhibited restrained apoptosis rates, with keloid FBs exhibiting greater restraint of apoptosis rates. It can be inferred that ASC restrained multiple bioactivities in keloid FBs and intensified normal FB functions. The scratch tests further proved hASCs' ability to promote wound healing primarily by enhancing normal FB functions, as opposed to keloid FB functions. We found that the cellular survival rates of normal and keloid FBs displayed no difference in normal-control groups. However, co-culture with hASCs exhibited a markedly suppressing effect on keloid FB survival, consistent with scratch test findings. The combination of the results above suggested that hASCs strongly suppressed keloid FB's proliferative ability and invasive behavior.

However, hASCs also regulated keloid FB's apoptosis negatively, which could counteract the suppressed survival rate. Overall, hASC interactions with normal and keloid FB cellular functions are beneficial to wound recovery and keloid improvement, despite the inhibition of keloid FB apoptosis.

Based on discoveries relating to cellular activities, we further tested Col-1, Col-3, CTGF, and P-4-HB, in terms of their effects on mRNA and protein levels. Collagen is the dominant protein of ECM 18 and is essential for cellular physiological function, including motility, metabolism, proliferation, differentiation, orientation, and survival. Fibrosis is a pathological process in the ECM leading to impairment of organ function 19. In traumatic skin, the synthesis of Col-3 even exceeds that of Col-1 in contrast to normal skin 20, 21. The dry weight ratio of Col-3 to Col-1 increases from approximately 1:4 in unlesioned dermis to 1:1 in injured dermis 4. CTGF is a member of the CCN protein family. It has been reported that CTGF can regulate multiple FB behaviors that can contribute to the development of fibrosis, including proliferation, differentiation, migration, adhesion, and matrix production. CTGF is highly expressed in various fibrotic conditions regulated by various profibrotic molecules, such as TGF-β_1_^22^, MAPK pathway 23, angiotensin II, and endothelin-1^24^. During fibrosis, FBs express excessive P-4-HB to stabilize collagen triple helix structure 25. Deactivation of P-4-HB reduces synthesis and secretion of pro-collagen Type Ⅰ and Ⅲ 26. Results from our qRT-PCR indicated that one of the pathways by which hASCs treated keloids was via stimulation of Col-1 expression in normal FBs. Another probable mechanism was suppression of P-4-HB expression in keloid FBs. Western blot results strongly demonstrated that hASCs could suppress keloids by inhibiting Col-3 and P-4-HB protein productions in keloid FBs. Intriguingly, in co-cultured groups, CTGF protein levels were also lower in keloid FBs than in normal FBs, which was not indicated by qRT-PCR. We assumed the alterations of CTGF synthesis were caused by regulation of its upstream molecules, which is worth further investigation.

Our current study proved hASCs' effectiveness in reversing keloid formation in accordance with the clinical outcome of autologous adipose tissue grafting. We found that hASCs regulated several cellular activities of normal or keloid FBs separately, from which we conjectured that keloid FBs were more of a target of hASCs than normal FBs. On the molecular level, our study involved two types of fibrosis-related genes: the ECM-related genes, including Col-1 and Col-3 genes and fibrosis markers, including CTGF and P-4-HB. Results revealed that ASC upregulated protein expression of Col-3 and CTGF in normal FBs but downregulated protein expression of CTGF and P-4-HB in keloid FBs, comprehensively demonstrated that hASCs contribute to keloid recovery by restricting several profibrotic molecules. Also new methods are expected for specific inhibition against hASCs' suppressing effect on the apoptosis rate of keloid FBs, because it would help further shorten the therapy period.

Consequently, ASCs not only promoted the bioactivities of normal FBs but also attenuated those of keloid FBs. As such, ASCs can be promising alternatives in both rejuvenation and keloid treatment.

## Supplementary Material

Supplementary figures.

## Figures and Tables

**Table 1:** Co-cultural scheme in a 24-well plate. A: adipose-derived stem cells; F: fibroblasts; NC: normal control; nFBs: normal fibroblasts; kFBs: keloid fibroblasts

	nFBs			kFBs		
	F1	F2	F3	F4	F5	F6
NC	F1+NC	F2+NC	F3+NC	F4+NC	F5+NC	F6+NC
A1	F1+A1	F2+A1	F3+A1	F4+A1	F5+A1	F6+A1
A2	F1+A2	F2+A2	F3+A2	F4+A2	F5+A2	F6+A2
A3	F1+A3	F2+A3	F3+A3	F4+A3	F5+A3	F6+A3
