# Supplementary material for: The Inhibition of Adipose-Derived Stem Cells on the Invasion of Keloid Fibroblasts
Source: Int J Med Sci. 2022 Oct 3;19(12):1796–805. doi: 10.7150/ijms.68646 (PMC9608046; doi:10.7150/ijms.68646)
Supplement: Supplementary file 1 — Supplementary figures. [file ijmsv19p1796s1.pdf]

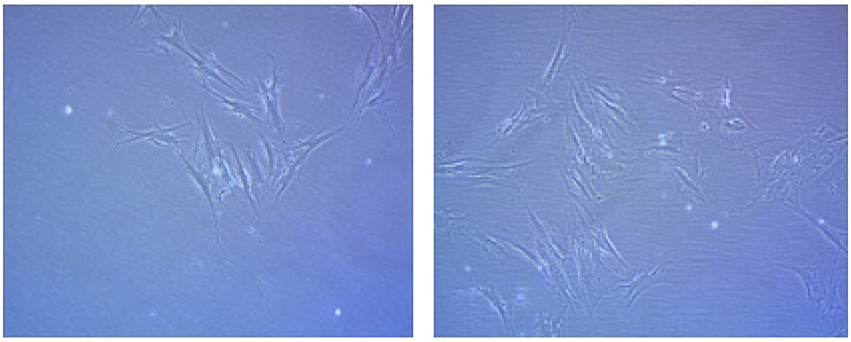

Supplementary Figure 1. Morphological features. hASCs are plastic-adherent. Original magnification is 100×. hASCs: human adipose-derived stem cells.

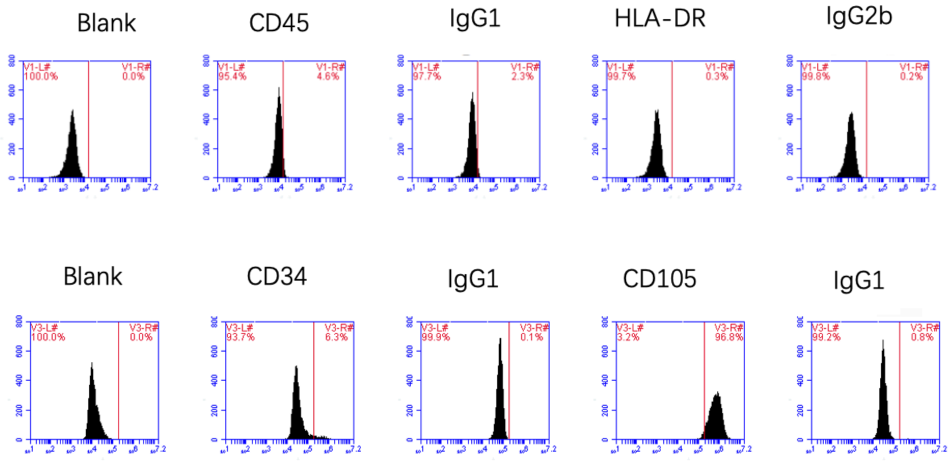

Supplementary Figure 2. The flow cytometry showed that the cells were positive for CD105 and negative for HLA-DR, CD45, and CD34.
